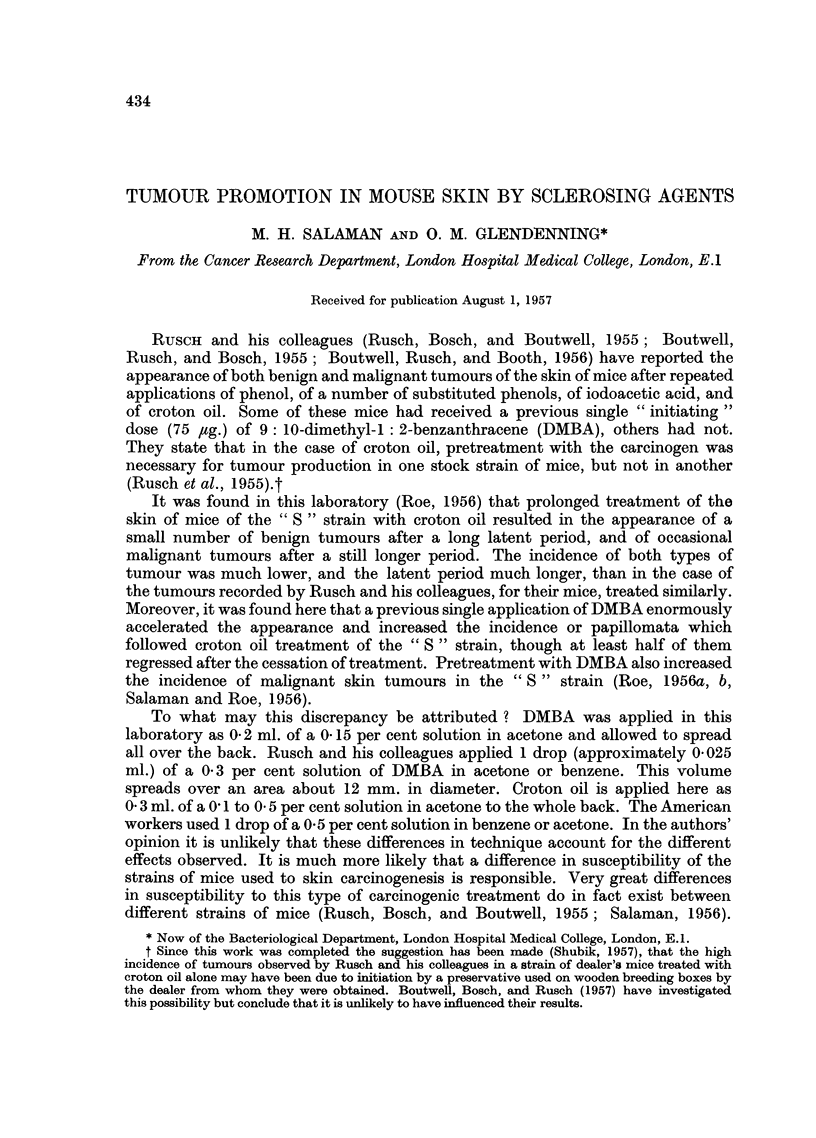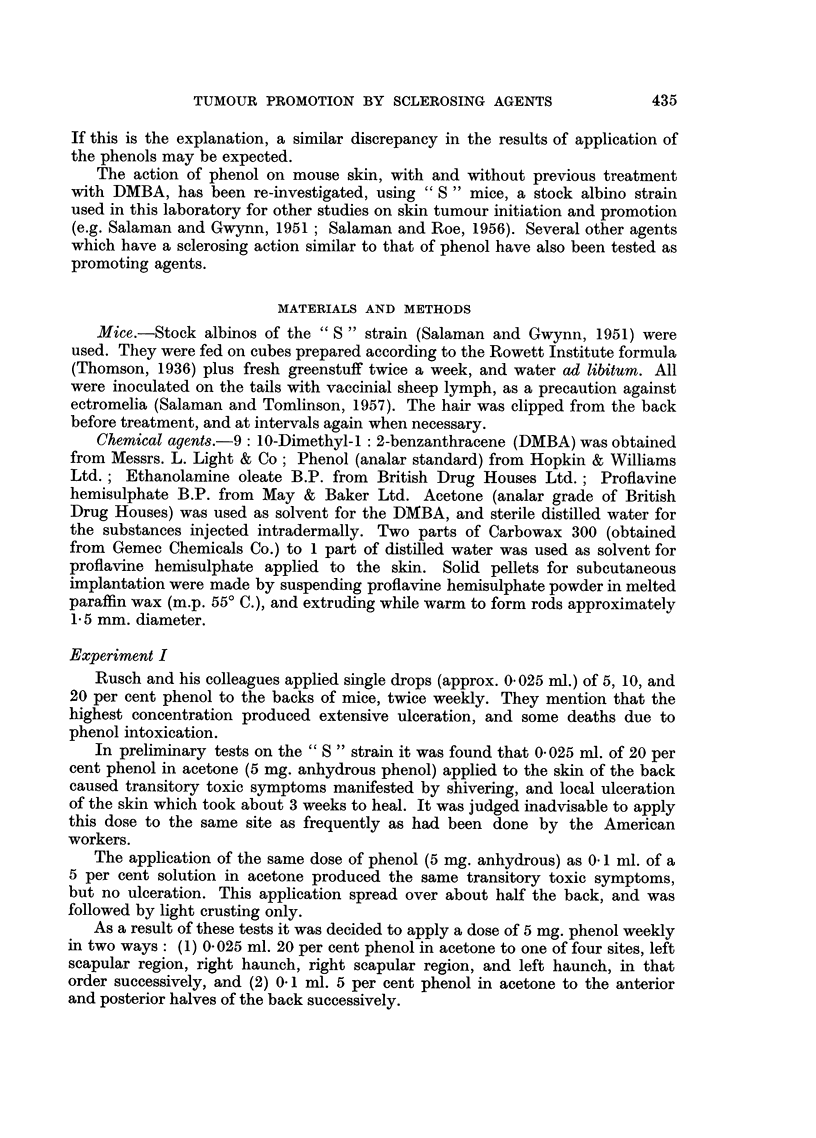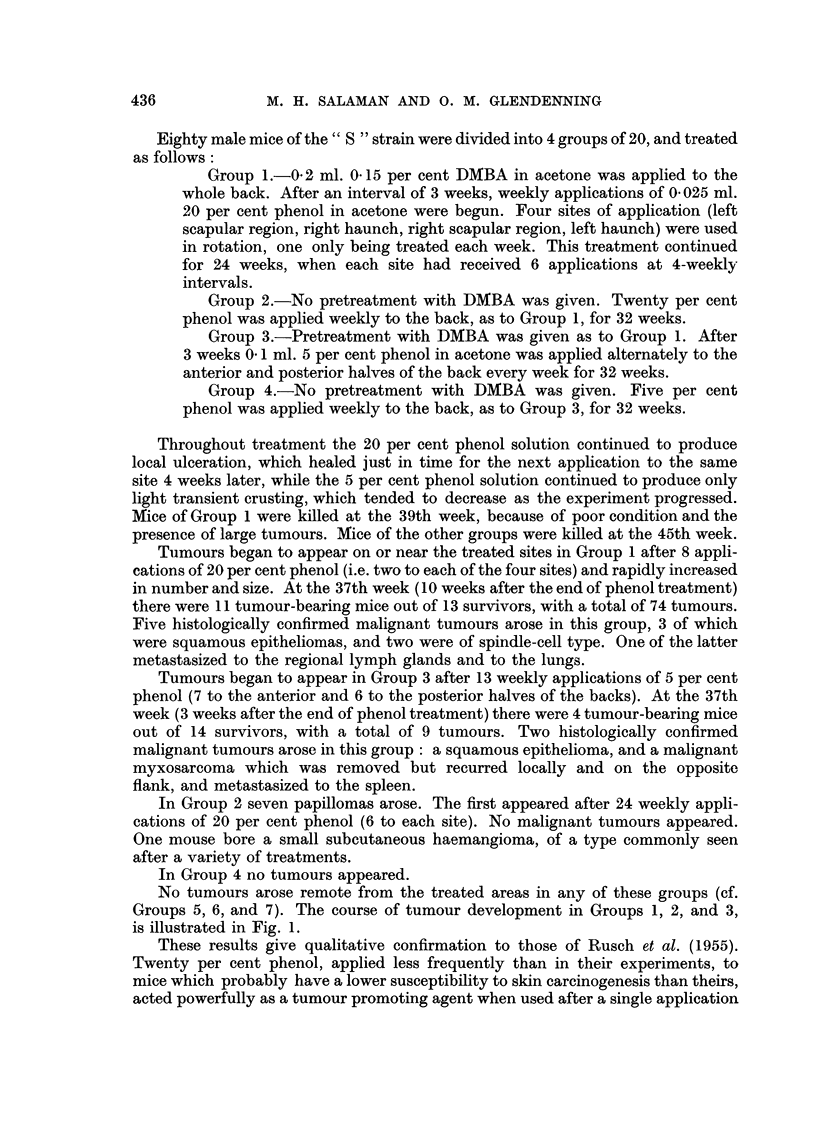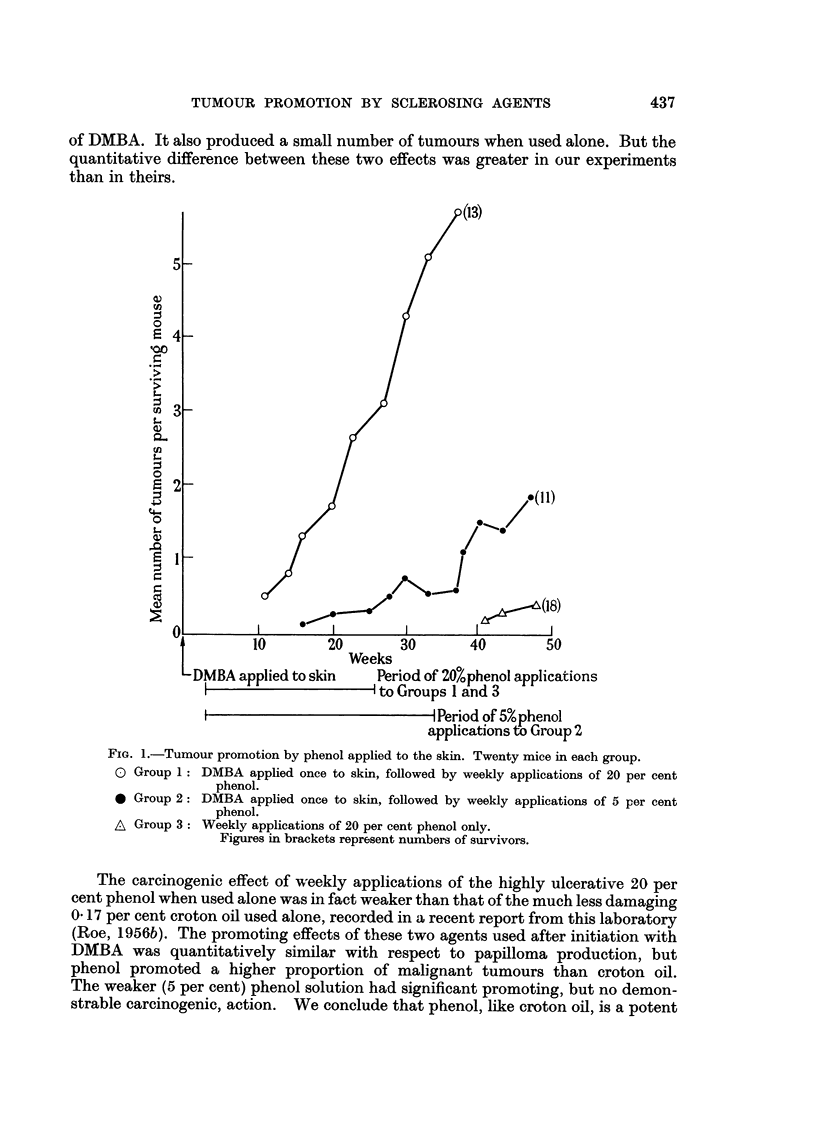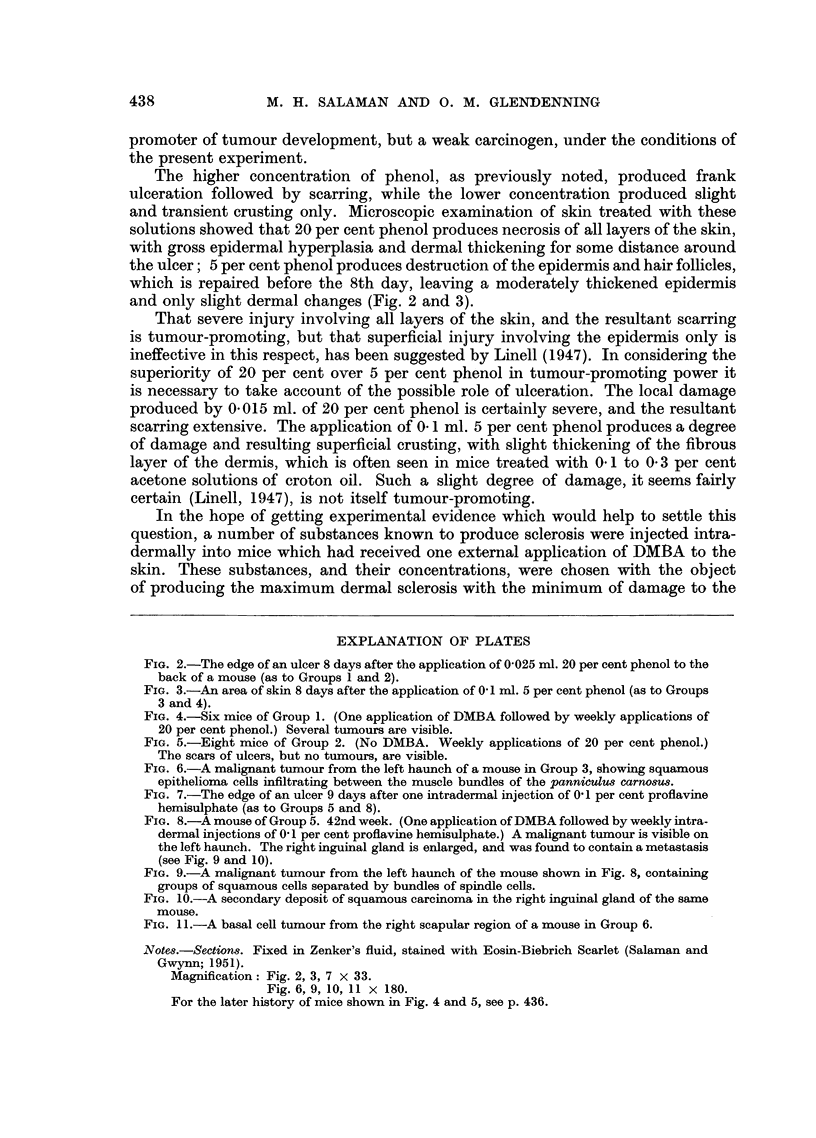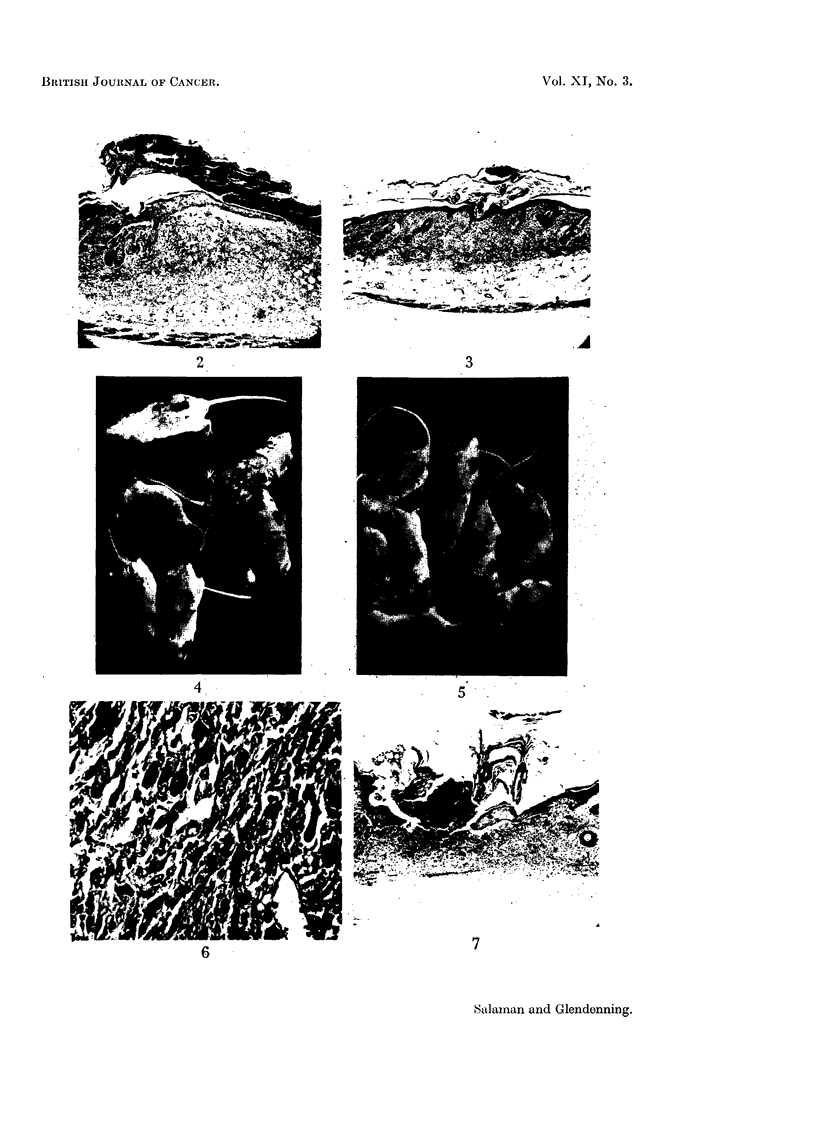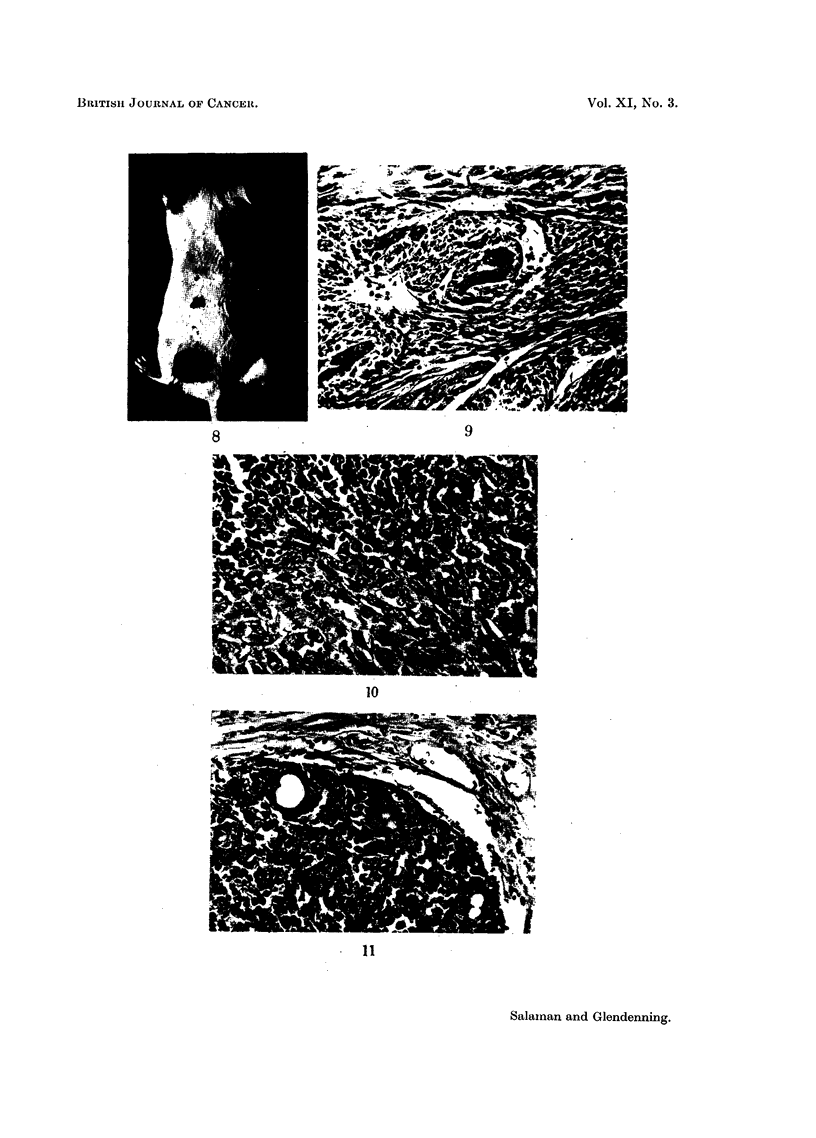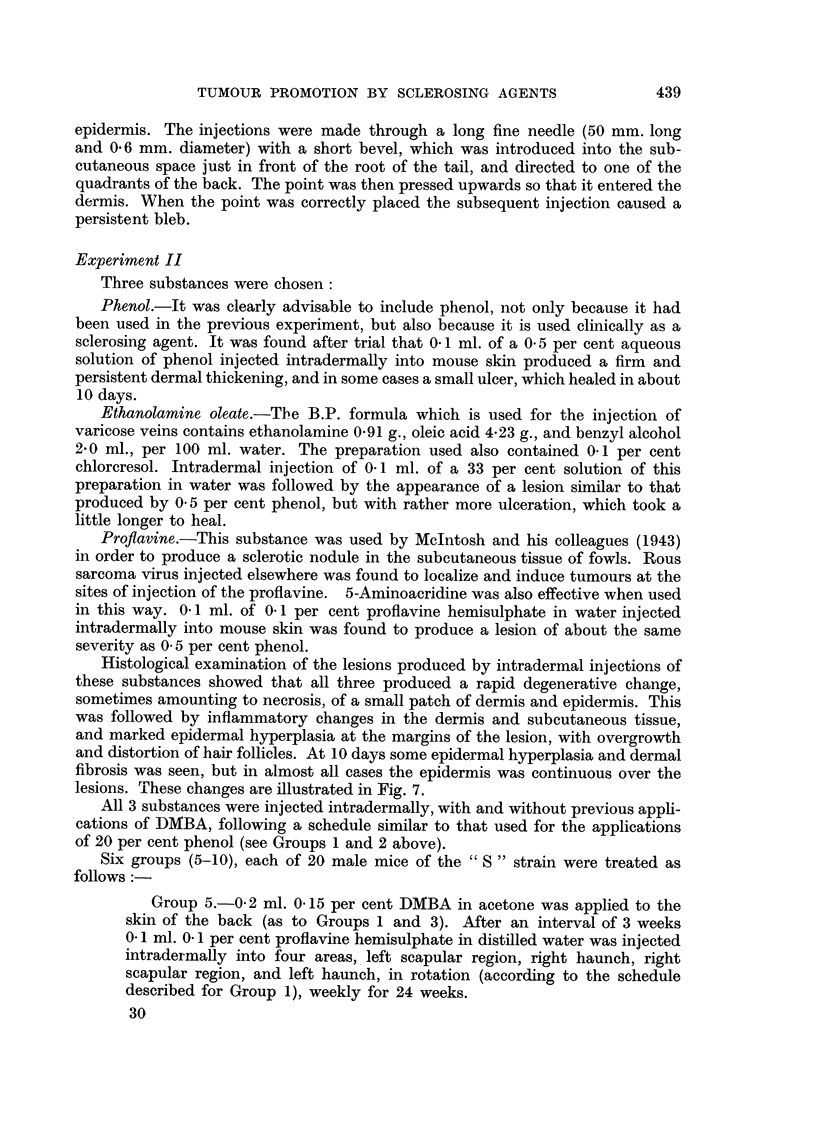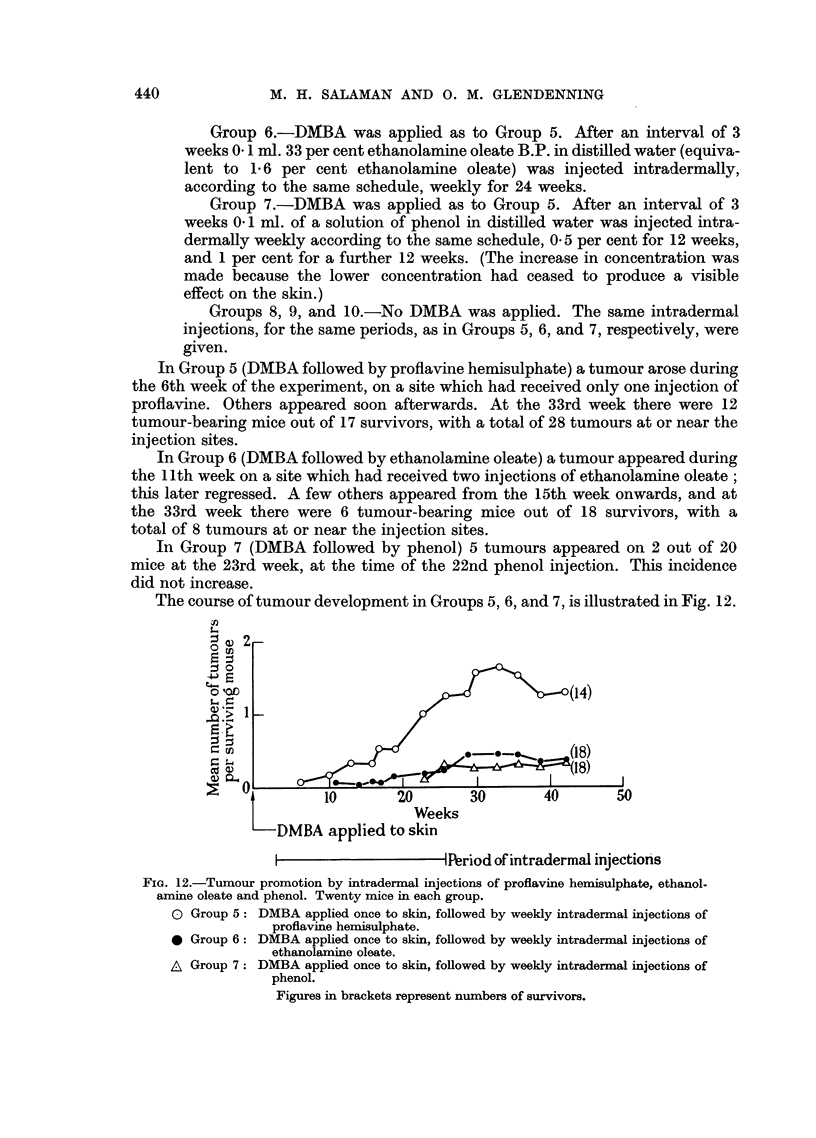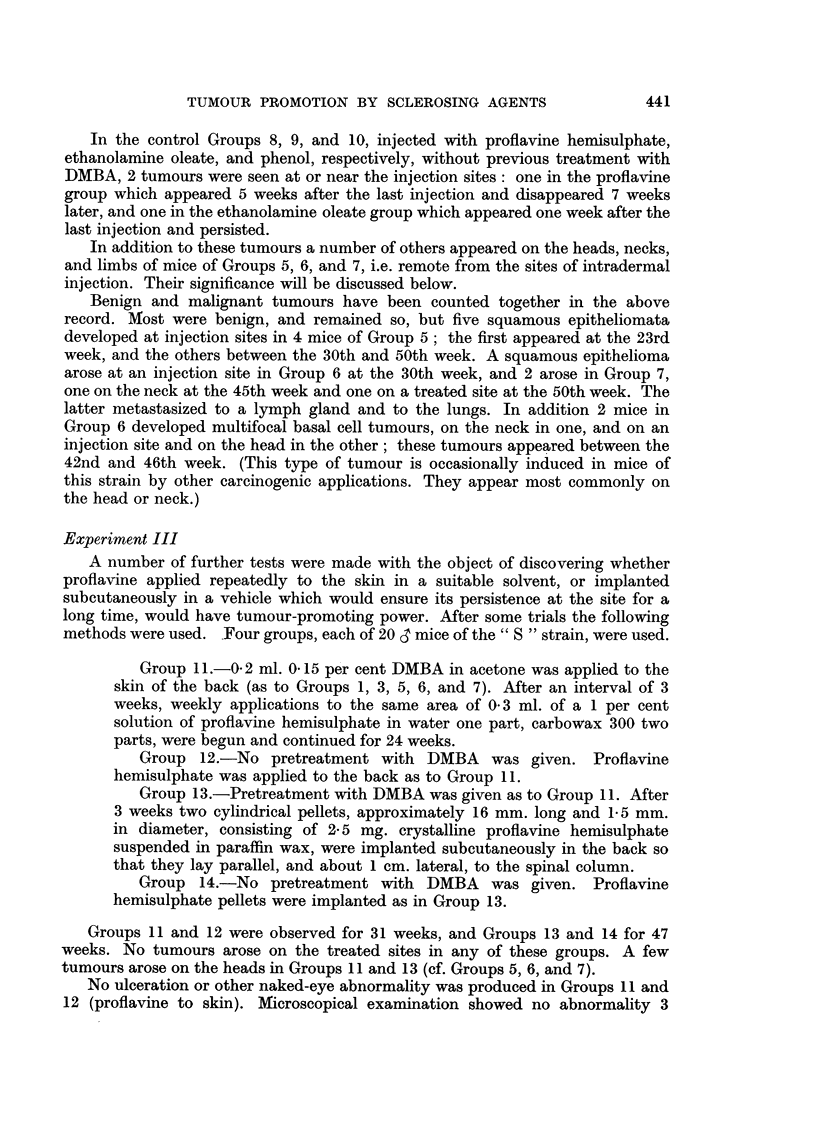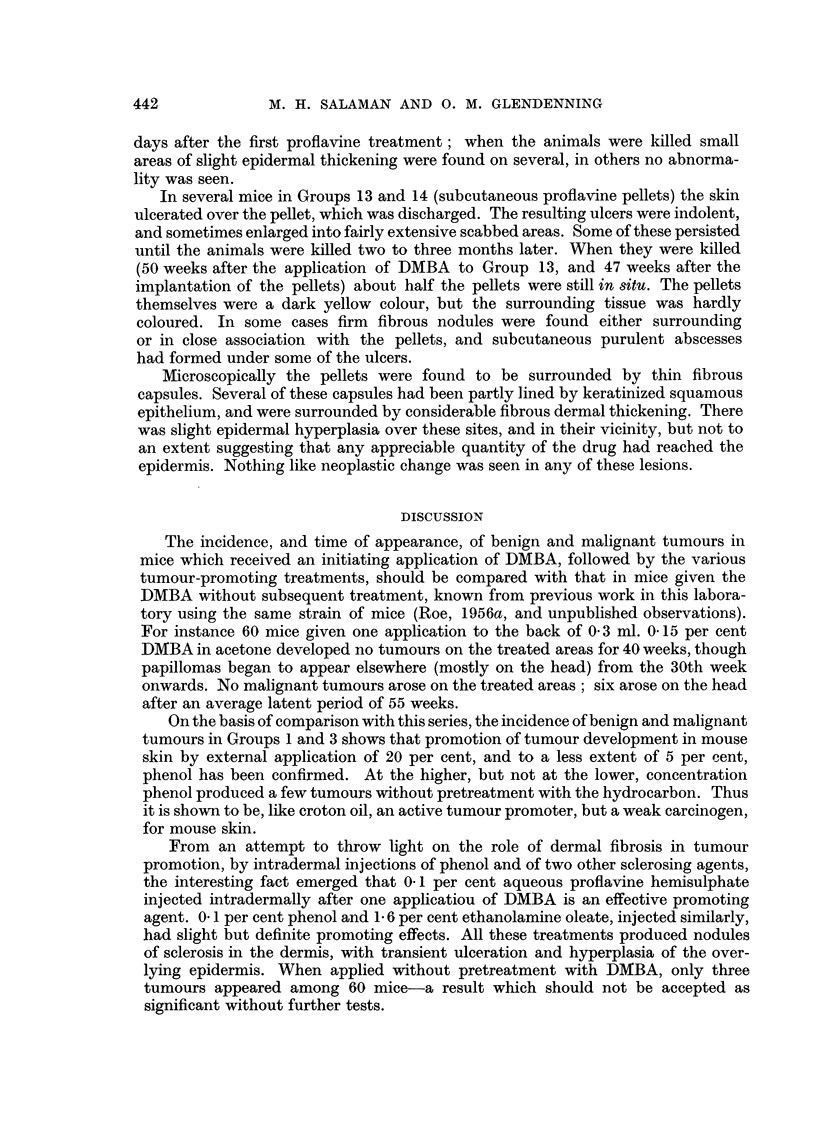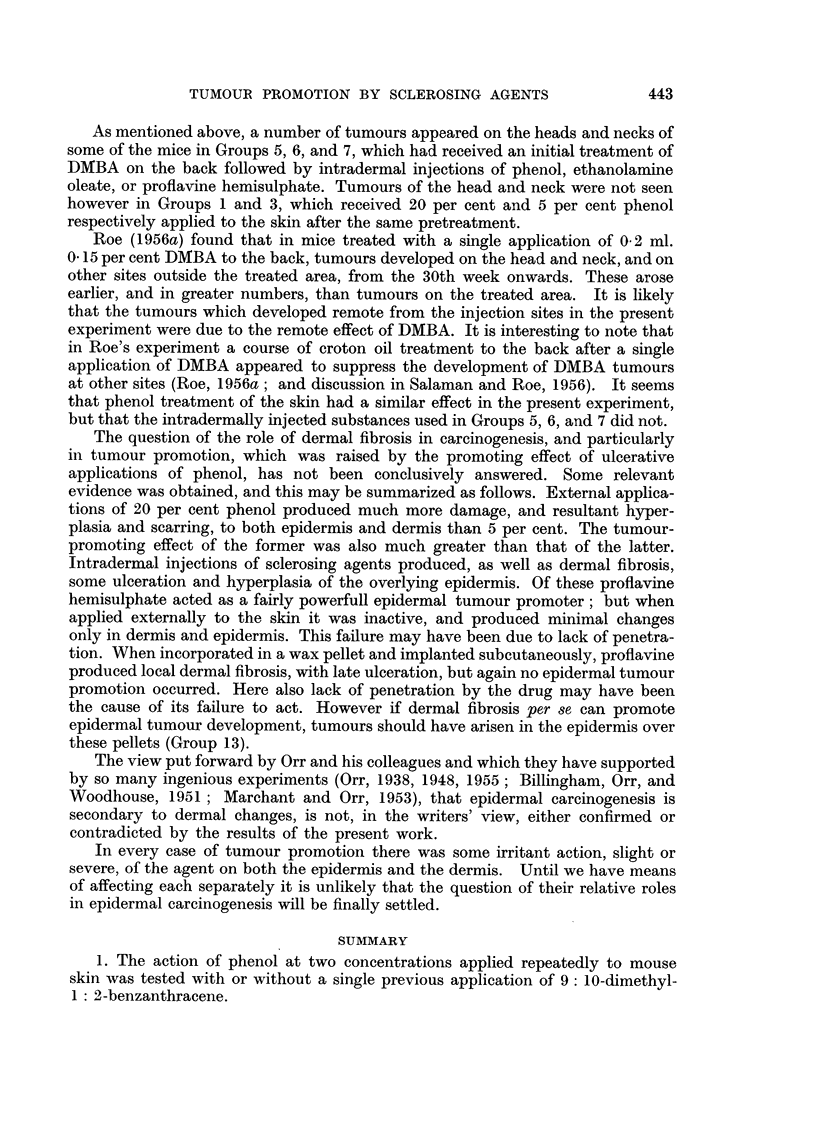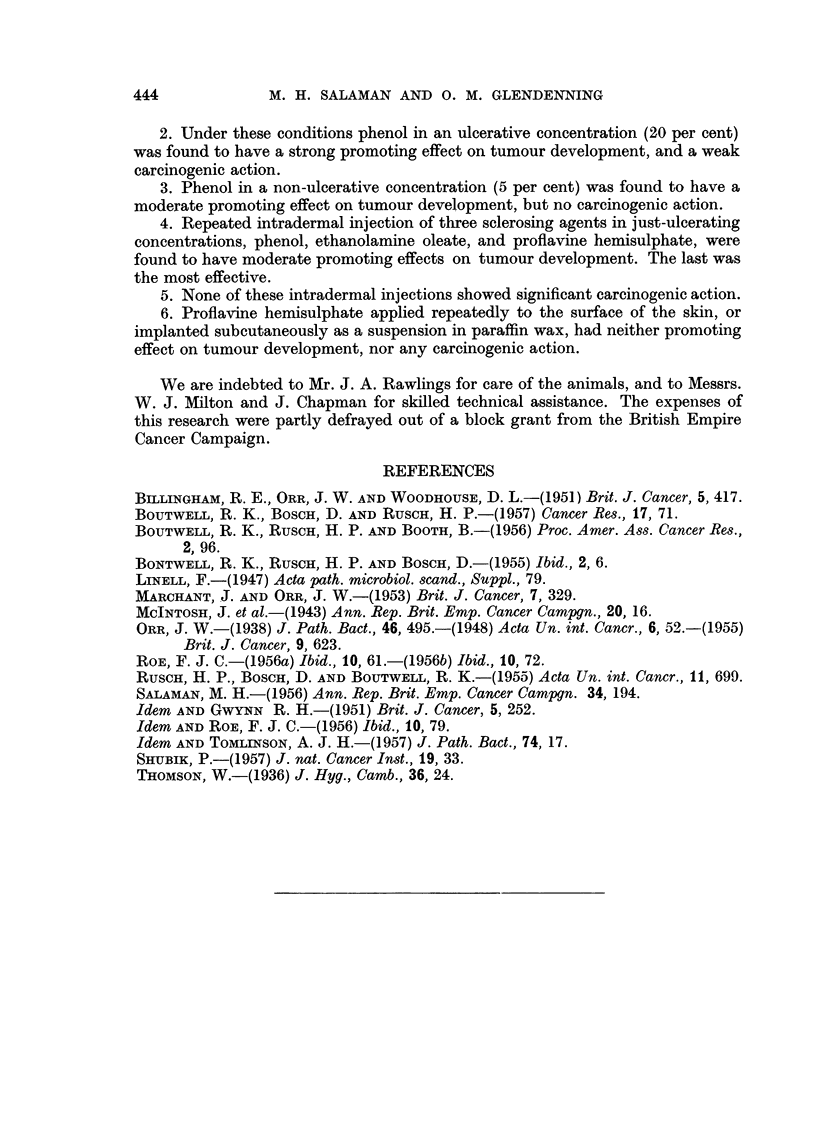# Tumour Promotion in Mouse Skin by Sclerosing Agents

**DOI:** 10.1038/bjc.1957.51

**Published:** 1957-09

**Authors:** M. H. Salaman, O. M. Glendenning

## Abstract

**Images:**


### TUMOUR PROMOTION IN MOUSE SKIN BY SCLEROSING AGENTS

M. H. SALAMAN AND O. M. GLENDENNING*

*From the Cancer Research Department, London Hospital Medical College, London, E.*1

Received for publication August 1, 1957

RUSCH and his colleagues (Rusch, Bosch, and Boutwell, 1955 ; Boutwell, Rusch, and Bosch, 1955 ; Boutwell, Rusch, and Booth, 1956) have reported the appearance of both benign and malignant tumours of the skin of mice after repeated applications of phenol, of a number of substituted phenols, of iodoacetic acid, and of croton oil. Some of these mice had received a previous single " initiating " dose (75 $\mu$g.) of 9 : 10-dimethyl-1 : 2-benzanthracene (DMBA), others had not. They state that in the case of croton oil, pretreatment with the carcinogen was necessary for tumour production in one stock strain of mice, but not in another (Rusch *et al.*, 1955).†

It was found in this laboratory (Roe, 1956) that prolonged treatment of the skin of mice of the " S " strain with croton oil resulted in the appearance of a small number of benign tumours after a long latent period, and of occasional malignant tumours after a still longer period. The incidence of both types of tumour was much lower, and the latent period much longer, than in the case of the tumours recorded by Rusch and his colleagues, for their mice, treated similarly. Moreover, it was found here that a previous single application of DMBA enormously accelerated the appearance and increased the incidence or papillomata which followed croton oil treatment of the " S " strain, though at least half of them regressed after the cessation of treatment. Pretreatment with DMBA also increased the incidence of malignant skin tumours in the " S " strain (Roe, 1956*a*, *b*, Salaman and Roe, 1956).

To what may this discrepancy be attributed ? DMBA was applied in this laboratory as 0·2 ml. of a 0·15 per cent solution in acetone and allowed to spread all over the back. Rusch and his colleagues applied 1 drop (approximately 0·025 ml.) of a 0·3 per cent solution of DMBA in acetone or benzene. This volume spreads over an area about 12 mm. in diameter. Croton oil is applied here as 0·3 ml. of a 0·1 to 0·5 per cent solution in acetone to the whole back. The American workers used 1 drop of a 0·5 per cent solution in benzene or acetone. In the authors' opinion it is unlikely that these differences in technique account for the different effects observed. It is much more likely that a difference in susceptibility of the strains of mice used to skin carcinogenesis is responsible. Very great differences in susceptibility to this type of carcinogenic treatment do in fact exist between different strains of mice (Rusch, Bosch, and Boutwell, 1955 ; Salaman, 1956).

* Now of the Bacteriological Department, London Hospital Medical College, London, E.1.

† Since this work was completed the suggestion has been made (Shubik, 1957), that the high incidence of tumours observed by Rusch and his colleagues in a strain of dealer's mice treated with croton oil alone may have been due to initiation by a preservative used on wooden breeding boxes by the dealer from whom they were obtained. Boutwell, Bosch, and Rusch (1957) have investigated this possibility but conclude that it is unlikely to have influenced their results.

If this is the explanation, a similar discrepancy in the results of application of the phenols may be expected.

The action of phenol on mouse skin, with and without previous treatment with DMBA, has been re-investigated, using " S " mice, a stock albino strain used in this laboratory for other studies on skin tumour initiation and promotion (e.g. Salaman and Gwynn, 1951 ; Salaman and Roe, 1956). Several other agents which have a sclerosing action similar to that of phenol have also been tested as promoting agents.

### MATERIALS AND METHODS

*Mice.*—Stock albinos of the " S " strain (Salaman and Gwynn, 1951) were used. They were fed on cubes prepared according to the Rowett Institute formula (Thomson, 1936) plus fresh greenstuff twice a week, and water *ad libitum.* All were inoculated on the tails with vaccinial sheep lymph, as a precaution against ectromelia (Salaman and Tomlinson, 1957). The hair was clipped from the back before treatment, and at intervals again when necessary.

*Chemical agents.*—9 : 10-Dimethyl-1 : 2-benzanthracene (DMBA) was obtained from Messrs. L. Light & Co ; Phenol (analar standard) from Hopkin & Williams Ltd. ; Ethanolamine oleate B.P. from British Drug Houses Ltd. ; Proflavine hemisulphate B.P. from May & Baker Ltd. Acetone (analar grade of British Drug Houses) was used as solvent for the DMBA, and sterile distilled water for the substances injected intradermally. Two parts of Carbowax 300 (obtained from Gemec Chemicals Co.) to 1 part of distilled water was used as solvent for proflavine hemisulphate applied to the skin. Solid pellets for subcutaneous implantation were made by suspending proflavine hemisulphate powder in melted paraffin wax (m.p. 55° C.), and extruding while warm to form rods approximately 1·5 mm. diameter.

### *Experiment I*

Rusch and his colleagues applied single drops (approx. 0·025 ml.) of 5, 10, and 20 per cent phenol to the backs of mice, twice weekly. They mention that the highest concentration produced extensive ulceration, and some deaths due to phenol intoxication.

In preliminary tests on the " S " strain it was found that 0·025 ml. of 20 per cent phenol in acetone (5 mg. anhydrous phenol) applied to the skin of the back caused transitory toxic symptoms manifested by shivering, and local ulceration of the skin which took about 3 weeks to heal. It was judged inadvisable to apply this dose to the same site as frequently as had been done by the American workers.

The application of the same dose of phenol (5 mg. anhydrous) as 0·1 ml. of a 5 per cent solution in acetone produced the same transitory toxic symptoms, but no ulceration. This application spread over about half the back, and was followed by light crusting only.

As a result of these tests it was decided to apply a dose of 5 mg. phenol weekly in two ways : (1) 0·025 ml. 20 per cent phenol in acetone to one of four sites, left scapular region, right haunch, right scapular region, and left haunch, in that order successively, and (2) 0·1 ml. 5 per cent phenol in acetone to the anterior and posterior halves of the back successively.

Eighty male mice of the " S " strain were divided into 4 groups of 20, and treated as follows :

Group 1.—0·2 ml. 0·15 per cent DMBA in acetone was applied to the whole back. After an interval of 3 weeks, weekly applications of 0·025 ml. 20 per cent phenol in acetone were begun. Four sites of application (left scapular region, right haunch, right scapular region, left haunch) were used in rotation, one only being treated each week. This treatment continued for 24 weeks, when each site had received 6 applications at 4-weekly intervals.

Group 2.—No pretreatment with DMBA was given. Twenty per cent phenol was applied weekly to the back, as to Group 1, for 32 weeks.

Group 3.—Pretreatment with DMBA was given as to Group 1. After 3 weeks 0·1 ml. 5 per cent phenol in acetone was applied alternately to the anterior and posterior halves of the back every week for 32 weeks.

Group 4.—No pretreatment with DMBA was given. Five per cent phenol was applied weekly to the back, as to Group 3, for 32 weeks.

Throughout treatment the 20 per cent phenol solution continued to produce local ulceration, which healed just in time for the next application to the same site 4 weeks later, while the 5 per cent phenol solution continued to produce only light transient crusting, which tended to decrease as the experiment progressed. Mice of Group 1 were killed at the 39th week, because of poor condition and the presence of large tumours. Mice of the other groups were killed at the 45th week.

Tumours began to appear on or near the treated sites in Group 1 after 8 applications of 20 per cent phenol (i.e. two to each of the four sites) and rapidly increased in number and size. At the 37th week (10 weeks after the end of phenol treatment) there were 11 tumour-bearing mice out of 13 survivors, with a total of 74 tumours. Five histologically confirmed malignant tumours arose in this group, 3 of which were squamous epitheliomas, and two were of spindle-cell type. One of the latter metastasized to the regional lymph glands and to the lungs.

Tumours began to appear in Group 3 after 13 weekly applications of 5 per cent phenol (7 to the anterior and 6 to the posterior halves of the backs). At the 37th week (3 weeks after the end of phenol treatment) there were 4 tumour-bearing mice out of 14 survivors, with a total of 9 tumours. Two histologically confirmed malignant tumours arose in this group : a squamous epithelioma, and a malignant myxosarcoma which was removed but recurred locally and on the opposite flank, and metastasized to the spleen.

In Group 2 seven papillomas arose. The first appeared after 24 weekly applications of 20 per cent phenol (6 to each site). No malignant tumours appeared. One mouse bore a small subcutaneous haemangioma, of a type commonly seen after a variety of treatments.

In Group 4 no tumours appeared.

No tumours arose remote from the treated areas in any of these groups (cf. Groups 5, 6, and 7). The course of tumour development in Groups 1, 2, and 3, is illustrated in Fig. 1.

These results give qualitative confirmation to those of Rusch *et al.* (1955). Twenty per cent phenol, applied less frequently than in their experiments, to mice which probably have a lower susceptibility to skin carcinogenesis than theirs, acted powerfully as a tumour promoting agent when used after a single application of DMBA. It also produced a small number of tumours when used alone. But the quantitative difference between these two effects was greater in our experiments than in theirs.

FIG. 1.—Tumour promotion by phenol applied to the skin. Twenty mice in each group.
○ Group 1 : DMBA applied once to skin, followed by weekly applications of 20 per cent phenol.
● Group 2 : DMBA applied once to skin, followed by weekly applications of 5 per cent phenol.
△ Group 3 : Weekly applications of 20 per cent phenol only.
Figures in brackets represent numbers of survivors.

The carcinogenic effect of weekly applications of the highly ulcerative 20 per cent phenol when used alone was in fact weaker than that of the much less damaging 0·17 per cent croton oil used alone, recorded in a recent report from this laboratory (Roe, 1956*b*). The promoting effects of these two agents used after initiation with DMBA was quantitatively similar with respect to papilloma production, but phenol promoted a higher proportion of malignant tumours than croton oil. The weaker (5 per cent) phenol solution had significant promoting, but no demonstrable carcinogenic, action. We conclude that phenol, like croton oil, is a potent promoter of tumour development, but a weak carcinogen, under the conditions of the present experiment.

The higher concentration of phenol, as previously noted, produced frank ulceration followed by scarring, while the lower concentration produced slight and transient crusting only. Microscopic examination of skin treated with these solutions showed that 20 per cent phenol produces necrosis of all layers of the skin, with gross epidermal hyperplasia and dermal thickening for some distance around the ulcer; 5 per cent phenol produces destruction of the epidermis and hair follicles, which is repaired before the 8th day, leaving a moderately thickened epidermis and only slight dermal changes (Fig. 2 and 3).

That severe injury involving all layers of the skin, and the resultant scarring is tumour-promoting, but that superficial injury involving the epidermis only is ineffective in this respect, has been suggested by Linell (1947). In considering the superiority of 20 per cent over 5 per cent phenol in tumour-promoting power it is necessary to take account of the possible role of ulceration. The local damage produced by 0·015 ml. of 20 per cent phenol is certainly severe, and the resultant scarring extensive. The application of 0·1 ml. 5 per cent phenol produces a degree of damage and resulting superficial crusting, with slight thickening of the fibrous layer of the dermis, which is often seen in mice treated with 0·1 to 0·3 per cent acetone solutions of croton oil. Such a slight degree of damage, it seems fairly certain (Linell, 1947), is not itself tumour-promoting.

In the hope of getting experimental evidence which would help to settle this question, a number of substances known to produce sclerosis were injected intradermally into mice which had received one external application of DMBA to the skin. These substances, and their concentrations, were chosen with the object of producing the maximum dermal sclerosis with the minimum of damage to the

---

EXPLANATION OF PLATES

FIG. 2.—The edge of an ulcer 8 days after the application of 0·025 ml. 20 per cent phenol to the back of a mouse (as to Groups 1 and 2).

FIG. 3.—An area of skin 8 days after the application of 0·1 ml. 5 per cent phenol (as to Groups 3 and 4).

FIG. 4.—Six mice of Group 1. (One application of DMBA followed by weekly applications of 20 per cent phenol.) Several tumours are visible.

FIG. 5.—Eight mice of Group 2. (No DMBA. Weekly applications of 20 per cent phenol.) The scars of ulcers, but no tumours, are visible.

FIG. 6.—A malignant tumour from the left haunch of a mouse in Group 3, showing squamous epithelioma cells infiltrating between the muscle bundles of the *panniculus carnosus*.

FIG. 7.—The edge of an ulcer 9 days after one intradermal injection of 0·1 per cent proflavine hemisulphate (as to Groups 5 and 8).

FIG. 8.—A mouse of Group 5. 42nd week. (One application of DMBA followed by weekly intradermal injections of 0·1 per cent proflavine hemisulphate.) A malignant tumour is visible on the left haunch. The right inguinal gland is enlarged, and was found to contain a metastasis (see Fig. 9 and 10).

FIG. 9.—A malignant tumour from the left haunch of the mouse shown in Fig. 8, containing groups of squamous cells separated by bundles of spindle cells.

FIG. 10.—A secondary deposit of squamous carcinoma in the right inguinal gland of the same mouse.

FIG. 11.—A basal cell tumour from the right scapular region of a mouse in Group 6.

*Notes.—Sections.* Fixed in Zenker's fluid, stained with Eosin-Biebrich Scarlet (Salaman and Gwynn; 1951).

Magnification: Fig. 2, 3, 7 × 33.

Fig. 6, 9, 10, 11 × 180.

For the later history of mice shown in Fig. 4 and 5, see p. 436.

2

3

4

5

6

7

8

9

10

11

Salaman and Glendenning.

epidermis. The injections were made through a long fine needle (50 mm. long and 0·6 mm. diameter) with a short bevel, which was introduced into the subcutaneous space just in front of the root of the tail, and directed to one of the quadrants of the back. The point was then pressed upwards so that it entered the dermis. When the point was correctly placed the subsequent injection caused a persistent bleb.

*Experiment II*

Three substances were chosen :

*Phenol.*—It was clearly advisable to include phenol, not only because it had been used in the previous experiment, but also because it is used clinically as a sclerosing agent. It was found after trial that 0·1 ml. of a 0·5 per cent aqueous solution of phenol injected intradermally into mouse skin produced a firm and persistent dermal thickening, and in some cases a small ulcer, which healed in about 10 days.

*Ethanolamine oleate.*—The B.P. formula which is used for the injection of varicose veins contains ethanolamine 0·91 g., oleic acid 4·23 g., and benzyl alcohol 2·0 ml., per 100 ml. water. The preparation used also contained 0·1 per cent chlorcresol. Intradermal injection of 0·1 ml. of a 33 per cent solution of this preparation in water was followed by the appearance of a lesion similar to that produced by 0·5 per cent phenol, but with rather more ulceration, which took a little longer to heal.

*Proflavine.*—This substance was used by McIntosh and his colleagues (1943) in order to produce a sclerotic nodule in the subcutaneous tissue of fowls. Rous sarcoma virus injected elsewhere was found to localize and induce tumours at the sites of injection of the proflavine. 5-Aminoacridine was also effective when used in this way. 0·1 ml. of 0·1 per cent proflavine hemisulphate in water injected intradermally into mouse skin was found to produce a lesion of about the same severity as 0·5 per cent phenol.

Histological examination of the lesions produced by intradermal injections of these substances showed that all three produced a rapid degenerative change, sometimes amounting to necrosis, of a small patch of dermis and epidermis. This was followed by inflammatory changes in the dermis and subcutaneous tissue, and marked epidermal hyperplasia at the margins of the lesion, with overgrowth and distortion of hair follicles. At 10 days some epidermal hyperplasia and dermal fibrosis was seen, but in almost all cases the epidermis was continuous over the lesions. These changes are illustrated in Fig. 7.

All 3 substances were injected intradermally, with and without previous applications of DMBA, following a schedule similar to that used for the applications of 20 per cent phenol (see Groups 1 and 2 above).

Six groups (5–10), each of 20 male mice of the " S " strain were treated as follows :—

Group 5.—0·2 ml. 0·15 per cent DMBA in acetone was applied to the skin of the back (as to Groups 1 and 3). After an interval of 3 weeks 0·1 ml. 0·1 per cent proflavine hemisulphate in distilled water was injected intradermally into four areas, left scapular region, right haunch, right scapular region, and left haunch, in rotation (according to the schedule described for Group 1), weekly for 24 weeks.

Group 6.—DMBA was applied as to Group 5. After an interval of 3 weeks 0·1 ml. 33 per cent ethanolamine oleate B.P. in distilled water (equivalent to 1·6 per cent ethanolamine oleate) was injected intradermally, according to the same schedule, weekly for 24 weeks.

Group 7.—DMBA was applied as to Group 5. After an interval of 3 weeks 0·1 ml. of a solution of phenol in distilled water was injected intradermally weekly according to the same schedule, 0·5 per cent for 12 weeks, and 1 per cent for a further 12 weeks. (The increase in concentration was made because the lower concentration had ceased to produce a visible effect on the skin.)

Groups 8, 9, and 10.—No DMBA was applied. The same intradermal injections, for the same periods, as in Groups 5, 6, and 7, respectively, were given.

In Group 5 (DMBA followed by proflavine hemisulphate) a tumour arose during the 6th week of the experiment, on a site which had received only one injection of proflavine. Others appeared soon afterwards. At the 33rd week there were 12 tumour-bearing mice out of 17 survivors, with a total of 28 tumours at or near the injection sites.

In Group 6 (DMBA followed by ethanolamine oleate) a tumour appeared during the 11th week on a site which had received two injections of ethanolamine oleate ; this later regressed. A few others appeared from the 15th week onwards, and at the 33rd week there were 6 tumour-bearing mice out of 18 survivors, with a total of 8 tumours at or near the injection sites.

In Group 7 (DMBA followed by phenol) 5 tumours appeared on 2 out of 20 mice at the 23rd week, at the time of the 22nd phenol injection. This incidence did not increase.

The course of tumour development in Groups 5, 6, and 7, is illustrated in Fig. 12.

Fig. 12.—Tumour promotion by intradermal injections of proflavine hemisulphate, ethanolamine oleate and phenol. Twenty mice in each group.

○ Group 5 : DMBA applied once to skin, followed by weekly intradermal injections of proflavine hemisulphate.

● Group 6 : DMBA applied once to skin, followed by weekly intradermal injections of ethanolamine oleate.

△ Group 7 : DMBA applied once to skin, followed by weekly intradermal injections of phenol.

Figures in brackets represent numbers of survivors.

In the control Groups 8, 9, and 10, injected with proflavine hemisulphate, ethanolamine oleate, and phenol, respectively, without previous treatment with DMBA, 2 tumours were seen at or near the injection sites : one in the proflavine group which appeared 5 weeks after the last injection and disappeared 7 weeks later, and one in the ethanolamine oleate group which appeared one week after the last injection and persisted.

In addition to these tumours a number of others appeared on the heads, necks, and limbs of mice of Groups 5, 6, and 7, i.e. remote from the sites of intradermal injection. Their significance will be discussed below.

Benign and malignant tumours have been counted together in the above record. Most were benign, and remained so, but five squamous epitheliomata developed at injection sites in 4 mice of Group 5 ; the first appeared at the 23rd week, and the others between the 30th and 50th week. A squamous epithelioma arose at an injection site in Group 6 at the 30th week, and 2 arose in Group 7, one on the neck at the 45th week and one on a treated site at the 50th week. The latter metastasized to a lymph gland and to the lungs. In addition 2 mice in Group 6 developed multifocal basal cell tumours, on the neck in one, and on an injection site and on the head in the other ; these tumours appeared between the 42nd and 46th week. (This type of tumour is occasionally induced in mice of this strain by other carcinogenic applications. They appear most commonly on the head or neck.)

*Experiment III*

A number of further tests were made with the object of discovering whether proflavine applied repeatedly to the skin in a suitable solvent, or implanted subcutaneously in a vehicle which would ensure its persistence at the site for a long time, would have tumour-promoting power. After some trials the following methods were used. Four groups, each of 20 ♂ mice of the " S " strain, were used.

Group 11.—0·2 ml. 0·15 per cent DMBA in acetone was applied to the skin of the back (as to Groups 1, 3, 5, 6, and 7). After an interval of 3 weeks, weekly applications to the same area of 0·3 ml. of a 1 per cent solution of proflavine hemisulphate in water one part, carbowax 300 two parts, were begun and continued for 24 weeks.

Group 12.—No pretreatment with DMBA was given. Proflavine hemisulphate was applied to the back as to Group 11.

Group 13.—Pretreatment with DMBA was given as to Group 11. After 3 weeks two cylindrical pellets, approximately 16 mm. long and 1·5 mm. in diameter, consisting of 2·5 mg. crystalline proflavine hemisulphate suspended in paraffin wax, were implanted subcutaneously in the back so that they lay parallel, and about 1 cm. lateral, to the spinal column.

Group 14.—No pretreatment with DMBA was given. Proflavine hemisulphate pellets were implanted as in Group 13.

Groups 11 and 12 were observed for 31 weeks, and Groups 13 and 14 for 47 weeks. No tumours arose on the treated sites in any of these groups. A few tumours arose on the heads in Groups 11 and 13 (cf. Groups 5, 6, and 7).

No ulceration or other naked-eye abnormality was produced in Groups 11 and 12 (proflavine to skin). Microscopical examination showed no abnormality 3

days after the first proflavine treatment ; when the animals were killed small areas of slight epidermal thickening were found on several, in others no abnormality was seen.

In several mice in Groups 13 and 14 (subcutaneous proflavine pellets) the skin ulcerated over the pellet, which was discharged. The resulting ulcers were indolent, and sometimes enlarged into fairly extensive scabbed areas. Some of these persisted until the animals were killed two to three months later. When they were killed (50 weeks after the application of DMBA to Group 13, and 47 weeks after the implantation of the pellets) about half the pellets were still *in situ.* The pellets themselves were a dark yellow colour, but the surrounding tissue was hardly coloured. In some cases firm fibrous nodules were found either surrounding or in close association with the pellets, and subcutaneous purulent abscesses had formed under some of the ulcers.

Microscopically the pellets were found to be surrounded by thin fibrous capsules. Several of these capsules had been partly lined by keratinized squamous epithelium, and were surrounded by considerable fibrous dermal thickening. There was slight epidermal hyperplasia over these sites, and in their vicinity, but not to an extent suggesting that any appreciable quantity of the drug had reached the epidermis. Nothing like neoplastic change was seen in any of these lesions.

### DISCUSSION

The incidence, and time of appearance, of benign and malignant tumours in mice which received an initiating application of DMBA, followed by the various tumour-promoting treatments, should be compared with that in mice given the DMBA without subsequent treatment, known from previous work in this laboratory using the same strain of mice (Roe, 1956*a*, and unpublished observations). For instance 60 mice given one application to the back of 0·3 ml. 0·15 per cent DMBA in acetone developed no tumours on the treated areas for 40 weeks, though papillomas began to appear elsewhere (mostly on the head) from the 30th week onwards. No malignant tumours arose on the treated areas ; six arose on the head after an average latent period of 55 weeks.

On the basis of comparison with this series, the incidence of benign and malignant tumours in Groups 1 and 3 shows that promotion of tumour development in mouse skin by external application of 20 per cent, and to a less extent of 5 per cent, phenol has been confirmed. At the higher, but not at the lower, concentration phenol produced a few tumours without pretreatment with the hydrocarbon. Thus it is shown to be, like croton oil, an active tumour promoter, but a weak carcinogen, for mouse skin.

From an attempt to throw light on the role of dermal fibrosis in tumour promotion, by intradermal injections of phenol and of two other sclerosing agents, the interesting fact emerged that 0·1 per cent aqueous proflavine hemisulphate injected intradermally after one applicatiou of DMBA is an effective promoting agent. 0·1 per cent phenol and 1·6 per cent ethanolamine oleate, injected similarly, had slight but definite promoting effects. All these treatments produced nodules of sclerosis in the dermis, with transient ulceration and hyperplasia of the overlying epidermis. When applied without pretreatment with DMBA, only three tumours appeared among 60 mice—a result which should not be accepted as significant without further tests.

As mentioned above, a number of tumours appeared on the heads and necks of some of the mice in Groups 5, 6, and 7, which had received an initial treatment of DMBA on the back followed by intradermal injections of phenol, ethanolamine oleate, or proflavine hemisulphate. Tumours of the head and neck were not seen however in Groups 1 and 3, which received 20 per cent and 5 per cent phenol respectively applied to the skin after the same pretreatment.

Roe (1956*a*) found that in mice treated with a single application of 0·2 ml. 0·15 per cent DMBA to the back, tumours developed on the head and neck, and on other sites outside the treated area, from the 30th week onwards. These arose earlier, and in greater numbers, than tumours on the treated area. It is likely that the tumours which developed remote from the injection sites in the present experiment were due to the remote effect of DMBA. It is interesting to note that in Roe's experiment a course of croton oil treatment to the back after a single application of DMBA appeared to suppress the development of DMBA tumours at other sites (Roe, 1956*a* ; and discussion in Salaman and Roe, 1956). It seems that phenol treatment of the skin had a similar effect in the present experiment, but that the intradermally injected substances used in Groups 5, 6, and 7 did not.

The question of the role of dermal fibrosis in carcinogenesis, and particularly in tumour promotion, which was raised by the promoting effect of ulcerative applications of phenol, has not been conclusively answered. Some relevant evidence was obtained, and this may be summarized as follows. External applications of 20 per cent phenol produced much more damage, and resultant hyperplasia and scarring, to both epidermis and dermis than 5 per cent. The tumour-promoting effect of the former was also much greater than that of the latter. Intradermal injections of sclerosing agents produced, as well as dermal fibrosis, some ulceration and hyperplasia of the overlying epidermis. Of these proflavine hemisulphate acted as a fairly powerfull epidermal tumour promoter ; but when applied externally to the skin it was inactive, and produced minimal changes only in dermis and epidermis. This failure may have been due to lack of penetration. When incorporated in a wax pellet and implanted subcutaneously, proflavine produced local dermal fibrosis, with late ulceration, but again no epidermal tumour promotion occurred. Here also lack of penetration by the drug may have been the cause of its failure to act. However if dermal fibrosis *per se* can promote epidermal tumour development, tumours should have arisen in the epidermis over these pellets (Group 13).

The view put forward by Orr and his colleagues and which they have supported by so many ingenious experiments (Orr, 1938, 1948, 1955 ; Billingham, Orr, and Woodhouse, 1951 ; Marchant and Orr, 1953), that epidermal carcinogenesis is secondary to dermal changes, is not, in the writers' view, either confirmed or contradicted by the results of the present work.

In every case of tumour promotion there was some irritant action, slight or severe, of the agent on both the epidermis and the dermis. Until we have means of affecting each separately it is unlikely that the question of their relative roles in epidermal carcinogenesis will be finally settled.

### SUMMARY

1. The action of phenol at two concentrations applied repeatedly to mouse skin was tested with or without a single previous application of 9 : 10-dimethyl-1 : 2-benzanthracene.

2. Under these conditions phenol in an ulcerative concentration (20 per cent) was found to have a strong promoting effect on tumour development, and a weak carcinogenic action.

3. Phenol in a non-ulcerative concentration (5 per cent) was found to have a moderate promoting effect on tumour development, but no carcinogenic action.

4. Repeated intradermal injection of three sclerosing agents in just-ulcerating concentrations, phenol, ethanolamine oleate, and proflavine hemisulphate, were found to have moderate promoting effects on tumour development. The last was the most effective.

5. None of these intradermal injections showed significant carcinogenic action.

6. Proflavine hemisulphate applied repeatedly to the surface of the skin, or implanted subcutaneously as a suspension in paraffin wax, had neither promoting effect on tumour development, nor any carcinogenic action.

We are indebted to Mr. J. A. Rawlings for care of the animals, and to Messrs. W. J. Milton and J. Chapman for skilled technical assistance. The expenses of this research were partly defrayed out of a block grant from the British Empire Cancer Campaign.

### REFERENCES

BILLINGHAM, R. E., ORR, J. W. AND WOODHOUSE, D. L.—(1951) *Brit. J. Cancer*, **5**, 417.
BOUTWELL, R. K., BOSCH, D. AND RUSCH, H. P.—(1957) *Cancer Res.*, **17**, 71.
BOUTWELL, R. K., RUSCH, H. P. AND BOOTH, B.—(1956) *Proc. Amer. Ass. Cancer Res.*, **2**, 96.
BONTWELL, R. K., RUSCH, H. P. AND BOSCH, D.—(1955) *Ibid.*, **2**, 6.
LINELL, F.—(1947) *Acta path. microbiol. scand., Suppl.*, 79.
MARCHANT, J. AND ORR, J. W.—(1953) *Brit. J. Cancer*, **7**, 329.
McINTOSH, J. *et al.*—(1943) *Ann. Rep. Brit. Emp. Cancer Campgn.*, **20**, 16.
ORR, J. W.—(1938) *J. Path. Bact.*, **46**, 495.—(1948) *Acta Un. int. Cancr.*, **6**, 52.—(1955) *Brit. J. Cancer*, **9**, 623.
ROE, F. J. C.—(1956*a*) *Ibid.*, **10**, 61.—(1956*b*) *Ibid.*, **10**, 72.
RUSCH, H. P., BOSCH, D. AND BOUTWELL, R. K.—(1955) *Acta Un. int. Cancr.*, **11**, 699.
SALAMAN, M. H.—(1956) *Ann. Rep. Brit. Emp. Cancer Campgn.* **34**, 194.
*Idem* AND GWYNN R. H.—(1951) *Brit. J. Cancer*, **5**, 252.
*Idem* AND ROE, F. J. C.—(1956) *Ibid.*, **10**, 79.
*Idem* AND TOMLINSON, A. J. H.—(1957) *J. Path. Bact.*, **74**, 17.
SHUBIK, P.—(1957) *J. nat. Cancer Inst.*, **19**, 33.
THOMSON, W.—(1936) *J. Hyg., Camb.*, **36**, 24.